# Comparison of Different Extraction Solvents for Characterization of Antioxidant Potential and Polyphenolic Composition in *Boletus edulis* and *Cantharellus cibarius* Mushrooms from Romania

**DOI:** 10.3390/molecules26247508

**Published:** 2021-12-11

**Authors:** Melinda Fogarasi, Maria-Ioana Socaciu, Claudiu-Dan Sălăgean, Floricuța Ranga, Anca Corina Fărcaș, Sonia Ancuța Socaci, Carmen Socaciu, Dorin Țibulcă, Szabolcs Fogarasi, Cristina Anamaria Semeniuc

**Affiliations:** 1 Department of Food Engineering, University of Agricultural Sciences and Veterinary Medicine Cluj-Napoca, 3-5 Mănăştur St., 400372 Cluj-Napoca, Romania; melinda.fogarasi@usamvcluj.ro (M.F.); maria-ioana.socaciu@usamvcluj.ro (M.-I.S.); dorin.tibulca@usamvcluj.ro (D.Ț.); 2 Department of Food Science, University of Agricultural Sciences and Veterinary Medicine Cluj-Napoca, 3-5 Mănăştur St., 400372 Cluj-Napoca, Romania; floricutza_ro@yahoo.com (F.R.); anca.farcas@usamvcluj.ro (A.C.F.); sonia.socaci@usamvcluj.ro (S.A.S.); carmen.socaciu@usamvcluj.ro (C.S.); 3 Faculty of Chemistry and Chemical Engineering, Babeş-Bolyai University, 11 Arany Janos Str., 400028 Cluj-Napoca, Romania; szabolcs.fogarasi@ubbcluj.ro

**Keywords:** mushrooms, *Boletus edulis*, *Cantharellus cibarius*, proximate composition, polyphenolic compounds, total phenolic content (TPC), total flavonoid content (TFC), Trolox equivalent antioxidant capacity (TEAC)

## Abstract

Edible mushrooms are well-known for their nutritional benefits and low energy density. In addition, mushroom extracts contain various bioactive compounds that account for their antioxidant activity; the applied extraction conditions influence the extraction efficiency of such compounds. Therefore, this study investigates the effects of four extractants on the content of polyphenols and antioxidant properties of *Boletus edulis* and *Cantharellus cibarius* mushrooms, aiming to optimize the extraction process. Powders of *B. edulis* and *C. cibarius* mushrooms were subjected to extraction with acidic water (10% CH_3_COOH), ethanol/water/acetic acid (15:76.5:8.5, *v*/*v*/*v*), hexane, and diethyl ether to measure their total phenolic content (TPC), total flavonoid content (TFC), and Trolox equivalent antioxidant capacity (TEAC). Furthermore, the level of individual polyphenolic compounds in these extracts was quantified using an HPLC-DAD-ESI-MS method. Results showed that the type of solvent significantly influenced the TPC and TEAC of mushroom powder but insignificantly influenced the TFC. A very strong positive correlation was found between TPC and TEAC, but no correlation was found between TFC and TEAC or TPC and TFC. Acidic water extracted the highest amount of polyphenolic compounds from these mushroom powders. Therefore, the aqueous extract showed the highest TPC and strongest antioxidant activity. Thus, acidic water is recommended for polyphenol analysis in *B. edulis* and *C. cibarius* mushrooms.

## 1. Introduction

The tradition of wild edible mushroom consumption has existed for centuries in many European countries [1]. Two hundred sixty-eight edible wild mushrooms are allowed for commercialization in Europe; the five top-selling edible fungi are *Boletus edulis*, *Cantharellus cibarius*, *Lactarius deliciosus*, *Morchella esculenta*, and *Agaricus campestris* [2]. *Boletus edulis* (porcini mushrooms) and *Cantharellus cibarius* (chanterelle) are among the most consumed wild mushrooms in Romania because of their availability in large quantities in this territory. These mushrooms are consumed fresh, dehydrated, or preserved in brine, vinegar, and oil.

Generally, mushrooms are characterized by high protein and carbohydrate content, and low-fat content [3]. In addition, mushrooms contain minerals [4,5] and bioactive compounds like phenols [3,6], flavonoids [3], ascorbic acid [3], *β*-carotene [3], and lycopene [7]. As is well-known, polyphenols, abundant in the fruiting bodies of mushrooms, have antioxidant properties [8]. Furthermore, the antioxidant activity of polyphenol-rich extracts was often correlated with total phenolic content (TPC) or total flavonoid content (TFC) [9,10], being influenced by the number and/or position of hydroxyl groups in phenolic and flavonoid compounds’ structure [11,12].

According to Singla et al. (2019), polyphenols are classified into flavonoids and non-flavonoids [13]. Flavonoids are subdivided into flavonols, flavones and isoflavones, anthocyanidins, and anthocyanins. Non-flavonoid compounds include phenolic acids, stilbenes, and lignans [13]. Among phenolic acids found in mushroom species were chlorogenic, gallic, caffeic, protocatechuic, and syringic acids. As well, representatives of flavonoids such as quercetin, hesperidin, and catechin were also reported [12].

Several studies have investigated the antioxidant capacity and phenols composition in different mushroom species. However, each study used another extraction method and/or other extraction solvents. Extraction solvents used in previous research included 50% ethanol [14], 60% ethanol [15], 70% ethanol [10], 80% ethanol [16], 95% ethanol [17], 96% ethanol [18], methanol/water (1:1, *v*/*v*) [18], 60% methanol [15,19], 70% methanol [10], 80% methanol [20,21], 95% methanol [17], pure methanol [3,9,22,23,24,25,26,27,28,29,30,31,32], methanol/chloroform (1:1, *v*/*v*) [33], 60% acetone [15], acetone/water (4:1, *v*/*v*) [34], acetone/water/acetic acid (70:29.5:0.5, *v*/*v*/*v*) [35], cyclohexane [29], dichloromethane [29], diethyl ether [14], hexane [15,36], water [3,10,14,15,17,26,29,30,31,37], and acidic water (1% HCl; 2 M HCl) [17,38]. Only a few of these reports focused on mushrooms from the species *B. edulis* [9,10,16,18,20,23,24,26] and *C. cibarius* [3,9,18,19,23,25,35,38]. Given the above-mentioned findings, there is a lack of studies on using acidic solvents to extract phenolic and antioxidant compounds from mushrooms. In addition, there is scarce information on the level of individual polyphenolic compounds in mushrooms extracts prepared with different solvents.

In the present work, an attempt has been made to evaluate the extraction efficiency of polyphenols from *Boletus edulis* and *Cantharellus cibarius* mushrooms with aqueous, hydro-alcoholic, hexanic, and etheric solvents (Aq, Aq-EtOH, Hex, Et2O). As far as we are aware, this is the first report investigating the effects of different extraction solvents [acidic water (10% CH_3_COOH), ethanol/water/acetic acid (15:76.5:8.5, *v*/*v*/*v*), hexane, and diethyl ether] on total phenolic content (TPC), total flavonoid content (TFC), Trolox equivalent antioxidant capacity (TEAC), and individual polyphenols of these mushroom powders. Our findings (data not shown) revealed that mixtures of water/acetic acid and ethanol/water/acetic acid, in the ratios mentioned above, proved effective in extracting plant polyphenolic compounds. Still, their extraction yields varied with the mushroom’s matrix. This study’s experimental design also included the characterization of powders obtained from these mushroom species by determining their proximate composition.

## 2. Results and Discussion

Proximate analysis results of *B. edulis* powder revealed a mean value of 26.5 ± 0.190% for protein content, 2.2 ± 0.058% for fat content, 53.8 ± 0.097% for total carbohydrate content, 7.1 ± 0.125% for ash content, and 10.4 ± 0.097% for moisture content. Therefore, the calculated energy value was 341 kcal/100 g mushroom powder. In *C. cibarius* powder a similar energy value (332 kcal/100 g) was found. However, a lower protein content (19.1 ± 0.066%), similar fat content (2.7 ± 0.068%), higher carbohydrate content (57.8 ± 0.123%), higher ash content (10.5 ± 0.116%), and similar moisture content (9.9 ± 0.010%) was noticed.

Table 1 shows values for total phenolic content (TPC), total flavonoid content (TFC), and Trolox equivalent antioxidant capacity (TEAC) of mushroom extracts. The highest TPC of *B. edulis* powder was found in Aq (3.73 mg GAE/g dw), followed by Aq-EtOH (3.42 mg GAE/g dw), Et2O (0.79 mg GAE/g dw), and Hex (0.48 mg GAE/g dw). For *C. cibarius* powder, the highest TPC was registered in Aq (0.79 mg GAE/g dw), followed by Aq-EtOH (0.66 mg GAE/g dw), Hex (0.58 mg GAE/g dw), and Et2O (0.29 mg GAE/g dw). Generally, total phenolic content strongly correlates with the antioxidant capacity [39]. A perfect positive relationship (*r* = 1.000; *p* < 0.001) between TPC and TEAC in *B. edulis* extracts was revealed, with Aq having the strongest antioxidant activity (1.90 µmol TE/g dw), followed by Aq-EtOH (1.79 µmol TE/g dw), Et2O (0.35 µmol TE/g dw), and Hex (0.23 µmol TE/g dw). In *C. cibarius* extracts, there was no significant correlation (*p* > 0.05) between TPC and TEAC; the highest TEAC value being noticed in Aq (1.12 µmol TE/g dw), followed by Aq-EtOH (1.00 µmol TE/g dw), Et2O (0.37 µmol TE/g dw), and Hex (0.28 µmol TE/g dw).

It is well known that each solvent has its specific selectivity towards the extraction of different compounds which contribute toward total antioxidant activity. For instance, acidic water provides the highest extraction efficiency, but at the same time, according to the results obtained by Wu et al. [40], it does not extract melanin very well. However, it is also important to note that even if the acidic water doesn’t extract melanin, it still gives the best performance compared to the other three solvents that manage to extract it. Still, these do not extract, or extract less than, other significant compounds which contribute to total antioxidant activity. Due to this diversity and variation of the extraction yield of different compounds, the performance of solvents is assessed based on the total antioxidant activity of extracts and not on the individual contribution of different compounds.

Unfortunately, it was difficult to compare our results to literature data because previous studies used different methods of preparing mushrooms, different extraction solvents and expressed results in other measurement units. To demonstrate this fact, several existing literature data on *B. edulis* and *C. cibarius* mushroom extracts were summarized in Table 2. These data indicate that, besides solvents used in the current study, there are other attractive options, like pure methanol, which gives very high yields regardless of the sample preparation method. Similarly, extraction with water proves to be very efficient in the case of freeze-dried mushroom powder. Comparing the findings of earlier reports (Table 2) to ours (Table 1), it appears that pure methanol and acetone/water/acid mixture provide better extraction performance than acidic water, the most efficient extractant among the four solvents used in this study. Still, acidic water would be the preferred solvent, considering that it is a green solvent and mushroom extracts are intended for food industry applications.

Regarding TFC, for *B. edulis* powder, the highest value was found in Aq (0.20 mg QUE/g dw), followed by Aq-EtOH (0.09 mg QUE/g dw), and equally by Hex (0.05 mg QUE/g dw) and Et2O (0.05 mg QUE/g dw). No significant correlations (*p* > 0.05) were detected between TFC and TEAC, respectively, between TPC and TFC in *B. edulis* extracts. In *C. cibarius* powder, the highest remarked TFC was in Et2O (0.30 mg QUE/g dw), followed by Aq-EtOH (0.28 mg QUE/g dw), Aq (0.03 mg QUE/g dw), and Hex (0.02 mg QUE/g dw). A very strong negative correlation (*r* = −0.980; *p* < 0.05) was observed between TFC and TEAC in *C. cibarius* extracts, but no significant one (*p* > 0.05) between TPC and TFC.

Generally, extraction solvent had a significant effect on TPC (*p* < 0.05) and TEAC (*p* < 0.001) in mushroom powder but not a significant one on TFC (*p* > 0.05). Values of TPC and TEAC in mushroom extracts were in the following order: aqueous > hydro-alcoholic > etheric > hexanic, hence the very strong positive correlation between TPC and TEAC (*r* = 0.897; *p* < 0.005). Although near to zero as values, the order of TFC in mushroom extracts was thusly: etheric > hexanic > aqueous > hydro-alcoholic; consequently, there were no significant correlations (*p* > 0.05) between TFC and TPC, respectively, or TFC and TEAC.

**Figure 1:**
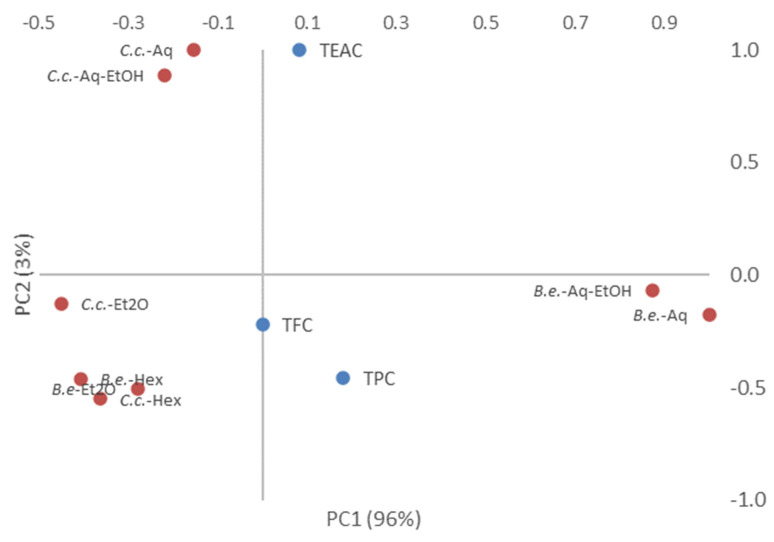
PCA biplot of mushroom extracts based on their TPC, TFC, and TEAC. *B.e.*-*Boletus edulis*; *C.c.*-*Cantharellus cibarius*; Aq-aqueous extract; Aq-EtOH-hydro-alcoholic extract; Hex-hexanic extract; Et2O-etheric extract; TPC-total phenolic content; TFC-total flavonoid content; TEAC-Trolox equivalent antioxidant capacity.

Principal component analysis (PCA) revealed an overall picture of mushroom extracts with regard to their strengths and weaknesses. A PCA biplot of mushroom extracts based on their TPCs, TFCs, and TEACs (Figure 1) display three main groups, divided based on similarities between them. The first principal component (PC1) explained 96% of the total variance, while the second (PC2) showed a further 3%. With the highest TPC and TEAC values, aqueous and hydro-alcoholic extracts of *B. edulis* powder were grouped in the lower-right quadrant. Seconding the first group as TPC and TEAC levels and having the lowest TFC values, Aq and Aq-EtOH of *C. cibarius* powder were plotted in the upper-left quadrant. Finally, hexanic and etheric extracts of *B. edulis* and *C. cibarius* powders were placed in the lower-left quadrant, with *C. cibarius* ones having the highest values of TFC.

Few studies have examined the polyphenolic profile in mushrooms of *B. edulis* [9,26,34] and *C. cibarius* [9,19,35,38]. Table 3 lists individual polyphenolic compounds quantified in *B. edulis* extracts from the current study. Seventeen compounds were identified in *B. edulis* mushrooms and grouped into two classes (phenolic acids-PAs and flavonoids-FVs) with five subclasses (hydroxybenzoic acids-HBAs, flavones-FVes, FVals-flavanols, flavonols-FVols, and hydroxycinnamic acids-HCAs). As a chemical class, the most abundant constituents in *B. edulis* powder were phenolic acids [87.4% in Aq (84.1% HBAs and 3.3% HCAs); 85.4% in Aq-EtOH (82.9% HBAs and 2.5% HCAs), 100% in Et2O (19.5% HBAs and 80.5% HCAs), and 100% in Hex (39.1% HBAs and 60.9% HCAs)], followed by flavonoids [12.6% in Aq (1.8% FVes, 4.2% FVals, and 6.6% FVols) and 14.6% in Aq-EtOH (1.8% FVes, 5.6% FVals, and 7.2% FVols)].

The major compounds identified in aqueous and hydro-alcoholic extracts of *B. edulis* powder were protocatechuic acid 4-*O*-glucoside (1735.35 µg/g dw in Aq and 1603.44 µg/g dw in Aq-EtOH), syringic acid (934.19 µg/g dw in Aq and 578.51 µg/g dw in Aq-EtOH), 2,4-dihydroxybenzoic acid (590.70 µg/g dw in Aq and 177.96 µg/g dw in Aq-EtOH), and gallic acid (371.46 µg/g dw in Aq and 235.06 µg/g dw in Aq-EtOH). All these compounds are hydroxybenzoic acids that belong to the phenolic acids class. Surprisingly, in the hexanic and etheric extracts of *B. edulis* powder, *trans*-cinnamic acid (39.71 µg/g dw in Hex and 54.45 µg/g dw in Et2O) and protocatechuic acid 4-*O*-glucoside (7.92 µg/g dw in Hex and 18.95 µg/g dw in Et2O) were predominant.

Palacios et al. (2011) reported a different profile in the methanolic extract of *B. edulis* mushrooms (see Table 2), with homogentisic acid, gallic acid, and protocatechuic acid as the main compounds [9]. In a study on methanolic extracts of *B. edulis* f. *beticola* and *B. edulis* f. *pinicola* mushrooms, Kuka and Cakste (2011) [26] discovered catechin as the principal compound of *B. edulis* f. *beticola*, respectively rutin followed by catechin as the majority compounds of *B. edulis* f. *pinicola*. Instead, in the hydro-acetonic extract of *B.edulis* mushrooms, Heleno et al. (2011) found higher amounts of *p*-hydroxybenzoic acid followed by cinnamic acid [34]. The study of Islam et al. (2016) evidenced gallic acid as the primary compound in the acidic hydro-acetonic extract of *C. cibarius* mushrooms [35].

Table 4 contains values of polyphenolic compounds detected in *C. cibarius* mushroom extracts. As with *B. edulis* ones, flavonoid compounds were not recovered by hexane and ether. Fourteen compounds were found in *C. cibarius* mushrooms. The predominant chemical constituents were phenolic acids [69.0% in Aq (69.0% HBAs); 63.7% in Aq-EtOH (63.7% HBAs), 100% in Et2O (100% HBAs), and 100% in Hex (100% HBAs)], followed by flavonoids [31.0% in Aq (6.6% FVals and 24.4% FVols) and 36.3% in Aq-EtOH (7.1% FVals and 29.2% FVols)].

The highest amounts noticed in aqueous and hydro-alcoholic extracts of *C. cibarius* powder were for 2,4-dihydroxybenzoic acid (202.48 µg/g dw in Aq and 90.33 µg/g dw in Aq-EtOH), protocatechuic acid 4-*O*-glucoside (179.90 µg/g dw in Aq and 253.54 µg/g dw in Aq-EtOH), syringic acid (166.69 µg/g dw in Aq and 171.60 µg/g dw in Aq-EtOH), epicatechin (153.81 µg/g dw in Aq and 194.39 µg/g dw in Aq-EtOH), gallic acid (112.51 µg/g dw in Aq and 63.37 µg/g dw in Aq-EtOH), catechin (111.94 µg/g dw in Aq and 115.46 µg/g dw in Aq-EtOH), and *p*-hydroxybenzoic acid (25.98 µg/g dw in Aq and 67.15 µg/g dw in Aq-EtOH). The most abundant compound in hexanoic and etheric extracts was protocatechuic acid 4-*O*-glucoside, of 5.40 µg/g dw and 20.67 µg/g dw, respectively.

Palacios et al. (2011) identified homogentisic acid, gallic acid, and pyrogallol as the main compounds in the methanolic extract of *C. cibarius* mushrooms [9]. Muszyńska et al. (2015) observed vanillic acid as the principal constituent in the acidic aqueous extract of *C. cibarius* powder, followed by *p*-hydroxybenzoic acid [38]. In the study of Islam et al. (2016) [35], gallic acid was the primary compound evidenced in the acid hydro-acetonic extract of *C. cibarius* mushrooms. Later, Butkhup et al. (2018) reported naringenin as the main compound in the hydro-methanolic extract of *C. cibarius* mushrooms, followed by catechin, epicatechin, and quercetin-3-*O*-rutinoside [19].

Comparing TPCs and TFCs of extracts resuspended in methanol to values of phenolic and flavonoid compounds of extracts resuspended in their extractants, the absence of flavonoid compounds was observed in hexanic and etheric extracts of the latter ones. Since values of flavonoid compounds spectrophotometrically assessed are close to zero in all extracts, both for *B. edulis* and *C. cibarius* mushroom powders, it can thus be deduced that these values were the result of measurement interferences. These findings are supported by the very strong positive relationship (*r* = 0.955; *p* < 0.001) found between phenolic compounds quantified in mushroom extracts by the HPLC method and the spectrophotometric method and the lack of a correlation (*p* > 0.05) between flavonoid compounds quantified by the two methods.

## 3. Materials and Methods

### 3.1. Solvents and Reagents

The following solvents and reagents were used: sodium sulphate anhydrous (VWR Chemicals, Leuven, Belgium), petroleum ether, boiling range 40–60 °C (Supelco Inc., Bellefonte, PA, USA), potassium sulfate (Supelco Inc., Bellefonte, PA, USA), copper(II) sulfate pentahydrate (Supelco Inc., Bellefonte, PA, USA), titanium(IV) oxide (Supelco Inc., Bellefonte, PA, USA), sulfuric acid 98% (Supelco Inc., Bellefonte, PA, USA), paraffin oil (Sigma-Aldrich Co., Saint Louis, MO, USA), boric acid (Supelco Inc., Bellefonte, PA, USA), bromocresol green (Supelco Inc., Bellefonte, PA, USA), methyl red (Supelco Inc., Bellefonte, PA, USA), sodium hydroxide (Supelco Inc., Bellefonte, PA, USA), sulfuric acid, 0.1 N volumetric solution (Supelco Inc., Bellefonte, PA, USA), pumice stone (Supelco Inc., Bellefonte, PA, USA), glacial acetic acid (Chempur, Sp. z o.o., Piekary Śląskie, Poland), ethanol, 95% (*v*/*v*) (Sigma-Aldrich Co., Saint Louis, MO, USA), hexane (Sigma-Aldrich Co., Saint Louis, MO, USA), diethyl ether (VWR Chemicals, Leuven, Belgium), methanol (VWR Chemicals, Fontenay-sous-Bois, France), gallic acid (Carl Roth GmbH + Co. KG, Karlsruhe, Germany), Folin-Ciocalteu’s phenol reagent (Sigma-Aldrich Co., Saint Louis, MO, USA), natriumcarbonat decahydrat (Carl Roth GmbH + Co. KG, Karlsruhe, Germany), aluminum chloride (Sigma-Aldrich Co., Saint Louis, MO, USA), sodium nitrite (Supelco Inc., Bellefonte, PA, USA), quercetin (Sigma-Aldrich Co., Saint Louis, MO, USA), 2,2-diphenyl-1-picrylhydrazyl (Thermo Fisher GmbH, Kandel, Germany), (R)-(+)-6-hydroxy-2,5,7,8-tetramethylchroman-2-carboxylic acid (Sigma-Aldrich Co., Saint Louis, MO, USA), polyamide syringe filters (Chromafil Xtra PA 45/25, Macherey-Nagel GmbH & Co. KG, Düren, Germany), glacial acetic acid, LC-MS grade (Supelco Inc., Bellefonte, PA, USA), ultrapure water (Supelco Inc., Bellefonte, PA, USA), acetonitrile, LC-MS grade (Supelco Inc., Bellefonte, PA, USA), rutin (Phytolab GmbH & Co. KG, Vestenbergsgreuth, Germany), catechin (Supelco Inc., Bellefonte, PA, USA), gallic acid (Phytolab GmbH & Co. KG, Vestenbergsgreuth, Germany), chlorogenic acid (Phytolab GmbH & Co. KG, Vestenbergsgreuth, Germany), methanol, LC-MS grade (Supelco Inc., Bellefonte, PA, USA), and nitrogen (SIAD România s.r.l., Bucharest, Romania).

### 3.2. Preparation of Mushroom Extracts

#### 3.2.1. Raw Materials

Two types of edible wild mushrooms, *Boletus edulis* and *Cantharellus cibarius*, were purchased in July 2021 from a local vegetable-fruit collection center (S.C. Alisa Funghi S.R.L., Chichisa, Romania). Mushrooms were manually sorted based on their size and appearance, cleaned of dirt and physical impurities, washed, drained, sliced, and dried (for 24 h at 45 °C) using a dehydrator (DEH-450, Biovita S.R.L., Cluj-Napoca, Romania). Dried mushrooms were further ground (GR-020, Minimoka, Paris, France); their powders were packed in amber glass jars with Teflon-lined caps and stored in a cool and dry place until analysis.

#### 3.2.2. Solvent Extraction

Four types of extracts were obtained from mushroom powders [Aq-aqueous extracts, Aq-EtOH-hydro-alcoholic extracts, Hex-hexanic extracts, and Et2O-etheric extracts] using the methods of Boonsong et al. (2016) and Sudha et al. (2016) with minor modifications [14,32].

The extraction process consisted of powder maceration with different solvents [acidic water-10% (*v*/*v*) acetic acid solution; a mixture of ethanol, water and acetic acid-15 parts of ethanol mixed with 76.5 parts distilled water and 8.5 parts acetic acid; hexane; and diethyl ether] as follows: homogenization of mushroom powder (5.0 g) with the extraction solvent (150 mL) at 21,500 rpm for 30 sec (T 18 digital Ultra-Turrax, IKA-Werke GmbH & Co. KG, Staufen, Germany), succeeded by orbital shaking at 150 rpm at room temperature for 24 h (MI01030002, Guangzhou Four E’s Scientific Co., Ltd., Guangzhou, China), centrifugation at 8981 × g (9000 rpm) at 4 °C for 10 min (Universal 320 R, Andreas Hettich GmbH & Co. KG, Tuttlingen, Germany), vacuum filtration of supernatant, removal of the solvent by rotary evaporation at 55 °C for Aq and Aq-EtOH respectively at 40 °C for Hex and Et2O (Hei-VAP Expert, Heidolph Instruments GmbH & Co. KG, Schwabach, Germany), and resuspension of dry extract in 10 mL extraction solvent (for HPLC-DAD-ESI-MS analysis of polyphenolic compounds) and 10 mL methanol (for spectrophotometric analysis of TPC, TFC and TEAC), respectively.

All extracts were stored at −18 °C until testing. These were filtered through polyamide syringe filters (0.45 µm pore size, 25 mm diameter) before chromatographic analysis and DPPH assay.

### 3.3. Determination of Mushroom Powders Proximate Composition

Moisture content was determined by sample drying to constant weight in an electric oven (Digitheat, J.P. Selecta S.A., Barcelona, Spain) following the instructions provided by ISO 712:2009 [41]. Samples were analyzed in triplicate. Results were expressed in percentage (%).

Protein content was determined using the Kjeldahl method described in ISO 20483:2013 [42]. It involved (1) acid digestion of the sample (DK6 Heating Digester, JP Recirculating Water Aspirator, VELP Scientifica S.R.L., Usmate Velate, Italy), (2) alkalization and steam distillation of acid digest (UDK 129 Distillation Unit, VELP Scientifica S.R.L., Usmate Velate, Italy), (3) and quantification of trapped ammonia by titration. First, the volume of 0.1 N sulfuric acid solution, used in titration, was used to calculate nitrogen content in the sample. Then, nitrogen content was multiplied by 4.38 to estimate the protein content [6]. Samples were analyzed in triplicate. Results were expressed in percentage (%).

Fat content was determined using the Soxhlet method described by Teklit (2015) [43], with minor modifications. A 1.0 g portion of the sample was weighed in a fat-free filter paper bag and loaded into a porous cellulose thimble. The thimble was immersed into an extraction cup (containing 80 mL petroleum ether) and placed into a Soxhlet extractor (SER 148 Solvent Extractor, VELP Scientifica S.R.L., Usmate Velate, Italy). The sample was extracted using the following instrument settings: hot plate temperature 110 °C, boiling time/sample immersion cycle time 30 min, sample rinse cycle time 4 h, solvent evaporation and recycle time 30 min. The extraction cup containing the extract was dried at 103 °C for 1 h in an electric oven (Digitheat, J.P. Selecta S.A., Barcelona, Spain), cooled at room temperature in a desiccator, and weighed with an analytical balance (ABJ-220-4NM, Kern & Sohn GmbH, Balingen, Germany). This step was repeated, at hourly intervals, until a constant weight was achieved. The fat content was calculated by the following Formula (1):

(1) $$\mathrm{Fat}\;\left(\%\right)=\frac{w_{f}}{w_{s}}\times 100$$

where $w_{f}$ is the weight of the fat (g) and $w_{s}$ is the weight of the sample (g). Samples were analyzed in triplicate.

Ash content was determined by sample incineration in a muffle furnace (L3/11/B170, Nabertherm GmbH, Bremen, Germany) according to the method described by Nagy et al. (2017) [44]. A 3.0 g portion of the sample was weighed and heated at 600 °C for 12 h in the muffle furnace. Next, it was cooled in a desiccator and weighed again. The ash content was calculated with the following Formula (2):

(2) $$\mathrm{Ash}\;\left(\%\right)=\frac{w_{a}}{w_{s}}\times 100$$

where $w_{a}$ is the weight of the ash (g) and $w_{s}$ is the weight of the sample (g). Samples were analyzed in triplicate.

Total carbohydrate content (%) was calculated by subtracting from 100 the sum of moisture, protein, fat, and ash contents (%) [44].

Energy value was calculated according to Nagy et al. (2017) [44], using the following Formula (3):

(3) Energy value (kcal/100 g)=4×(g protein+g carbohydrate)+9×(g fat)

### 3.4. Determination of Total Phenolic Content

Total phenolic content in mushroom extracts was determined using the method described by Semeniuc et al. (2018) [45]. One hundred microliters of the extract were transferred into a 16-mL glass bottle with a rubber stopper; 6 mL distilled water, and 0.5 mL of 2 N Folin-Ciocalteu’s phenol reagent were then added and vortexed (Vortex V-1 Plus, Biosan Ltd., Riga, Latvia). After 4 min, 1.5 mL of 0.71 M sodium carbonate solution and 1.9 mL distilled water were added and vortexed again. The mixture was kept in the dark, at room temperature, for 2 h. The absorbance value of the mixture was read at 725 nm against the blank using a double beam UV-VIS spectrophotometer (UV-1900i, Shimadzu Scientific Instruments, Inc., Columbia, MD, USA). The blank was prepared with methanol (instead of extract) and treated identically to the test sample. Samples were tested in triplicate. For the calibration curve preparation, gallic acid was used in concentrations from 0.25 to 1.25 mg/mL. Results were expressed in mg gallic acid equivalents (GAE)/g dry weight (dw).

### 3.5. Determination of Total Flavonoid Content

Determination of total flavonoid in mushroom extract content was carried out as previously described by Socaci et al. (2018) [46]. One hundred microliters of the extract were diluted to a final volume of 5 mL with distilled water and then transferred into a 16-mL glass bottle with a rubber stopper. Next, 300 µL of 5% (*w*/*v*) sodium nitrite solution were added and vortexed (Vortex V-1 Plus, Biosan Ltd., Riga, Latvia). After 5 min, 300 µL of 10% (*w*/*v*) aluminum chloride solution, and after another 5 min, 2 mL of 1 N sodium hydroxide solution were added and mixed using the vortex. The absorbance value of the mixture was read at 510 nm against the blank using a double beam UV-VIS spectrophotometer (UV-1900i, Shimadzu Scientific Instruments, Inc., Columbia, MD, USA). The blank was prepared with methanol (instead of aluminum chloride solution) and treated identically to the test sample. Samples were tested in triplicate. For the calibration curve preparation, quercetin was used in concentrations from 0.019 to 0.800 mg/mL. Results were expressed in mg quercetin equivalents (QUE)/g dry weight (dw).

### 3.6. Determination of Antioxidant Capacity by DPPH (2,2-Diphenyl-1-picrylhydrazyl) Assay

DPPH assay was performed as described by Thaipong et al. (2006) [47]. First, a stock solution was prepared by dissolving 24 mg DPPH in 100 mL methanol. Then, 24 mL of stock solution was mixed with 90 mL methanol to reach an absorbance of 1.1 ± 0.02 units at 515 nm, thus obtaining the working solution.

One hundred fifty microliters of the extract were transferred into a 16-mL glass bottle with a rubber stopper; 2850 μL of DPPH working solution was then added and vortexed (Vortex V-1 Plus, Biosan Ltd., Riga, Latvia). The mixture was kept in the dark, at room temperature, for 1 h. The absorbance value of the mixture was read at 515 nm against methanol using a double-beam UV-VIS spectrophotometer (UV-1900i, Shimadzu Scientific Instruments, Inc., Columbia, MD, USA). The blank was prepared with methanol (instead of extract) and treated identically to the test sample. The absorbance value of the blank was subtracted from that of the extract. Samples were tested in triplicate. For the calibration curve preparation, Trolox was used in concentrations from 25 to 800 µmol/L. Results were expressed in µM Trolox equivalent (TE)/g dry weight (dw).

### 3.7. Identification and Quantification of Polyphenolic Compounds by HPLC-DAD-ESI-MS

The HPLC-DAD-ESI-MS procedure published by Călinoiu and Vodnar (2020) [48] was used. Separation, identification, and quantification of individual polyphenolic compounds were performed on a liquid chromatography system (1200 HPLC, Agilent Technologies Inc., Palo Alto, CA, USA). It contained a photodiode array (PDA) detector (G1315B), a single-quadrupole mass spectrometer (MS) (G6110) equipped with an electrospray ionization (ESI) source (G1948B). The system also included a quaternary pump (G1311A), a degasser (G1322A), an autosampler (G1329A), a thermostatted column compartment (G1316A), a Kinetex XB-C18 column (150 mm L × 4.6 mm ID × 5 μm particle size; Phenomenex, Torrance, CA, USA), and the ChemStation software (Rev B.04.02 SP1, Agilent Technologies Inc., Palo Alto, CA, USA).

Twenty microliters of extract were injected into the HPLC system for analysis. In the elution process, two mobile phases were employed: solution A containing 0.1% acetic acid in ultrapure water, and solution B containing 0.1% acetic acid in acetonitrile. A multi-step gradient elution model was used with the following settings: 5% B (0–2 min), from 5 to 90% B (2–20 min), 90% B (20–24 min), from 90 to 5% B (24–30 min). The flow rate was programmed to 0.5 mL/min and the column oven temperature to 25 °C. The PDA was set to scan from 200 to 600 nm. The chromatograms were acquired at 280 and 340 nm; data acquisition was performed for 30 min.

Mass spectra were recorded using the ESI source set in positive-ion mode and the MS in full scan mode (in an *m*/*z* range of 120−1200). Nitrogen was used as a drying gas. Other settings for the ESI source were as follows: drying gas temperature 350 °C, drying gas flow rate 7 L/min, nebulizer pressure 35 psi, capillary voltage 3000 V, fragmentor voltage 100 eV.

Polyphenolic compounds were tentatively identified by comparing their retention times, UV-vis spectra, and mass spectra to those of the standards analyzed under the same conditions and the data available in the literature [49,50].

The standards of rutin, catechin, gallic acid, and chlorogenic acid were prepared with methanol as solvent. Flavones and flavonols were quantified using a five-point analytical curve of rutin (10−100 µg/mL; *r*^2^ = 0.9981); flavanols using a five-point analytical curve of catechin (10−100 µg/mL; *r*^2^ = 0.9994); hydroxybenzoic acids using a five-point analytical curve of gallic acid (5−100 µg/mL; *r*^2^ = 0.9978); and hydroxycinnamic acids using a five-point analytical curve of chlorogenic acid (10−50 µg/mL; *r*^2^ = 0.9937). Samples were tested in triplicate. Results were expressed in µg/g dry weight (dw).

### 3.8. Statistical Analysis

For data analysis, was used Minitab statistical software (version 19.1.1, LEAD Technologies, Inc., Charlotte, NC, USA). First, the differences between the four treatments [(1) extraction with acidic water (10% CH_3_COOH), (2) extraction with ethanol/water/acetic acid (15:76.5:8.5, *v*/*v*/*v*), (3) extraction with hexane, and (4) extraction with diethyl ether)] were determined using one-way ANOVA. In addition, was performed post-hoc pairwise comparisons with Tukey’s test at a 95% confidence level (*p* < 0.05). Then, the strength of relationships between TPC and TEAC, TFC and TEAC, or TPC and TFC was measured by calculating Pearson’s correlation coefficients (*r*). When the value of *r* is in-between 0.0–0.19, the correlation is very weak; weak between 0.20–0.39, moderate between 0.40–0.59, strong between 0.60–0.79, and very strong between 0.80–1.0. The significance of the correlation was interpreted based on the *p*-value as follows: *p* ≥ 0.05^NS^, not significant trend; 0.01 ≤ *p* < 0.05, weakly significant; 0.005 ≤ *p* < 0.01, mildly significant; 0.001 ≤ *p* < 0.005, moderately significant; *p* < 0.001, strongly significant. Finally, principal component analysis (PCA) was carried out using Unscrambler software (version 9.7, CAMO Software AS, Oslo, Norway).

## 4. Conclusions

This study reveals that *B. edulis* and *C. cibarius* mushrooms are sources of polyphenolic compounds and exhibit antioxidant activities. However, each type of tested solvent provided different extraction yields of phenolic and flavonoid compounds and different antioxidant responses. For example, mixtures of water/acetic acid (9:1, *v*/*v*) and ethanol/water/acetic acid (15:76.5:8.5, *v*/*v*/*v*) extracted higher amounts of polyphenolic compounds than ether or hexane and demonstrated better antioxidant activities. Therefore, acidic water (10% CH_3_COOH) is the recommended solvent for extracting these bioactive compounds from *B. edulis* and *C. cibarius* mushrooms.

## Figures and Tables

**Table 1:** Total phenolic contents (mg GAE/g dw), total flavonoid contents (TFC mg QUE/g dw), and antioxidant activities (µmol TE/g dw) of mushroom extracts.

Mushrooms Powder of:	Type of Extract	TPC	TFC	TEAC
*B. edulis*	Aq	3.73 ± 0.007 ^a^	0.20 ±0.002 ^a^	1.90 ± 0.003 ^a^
	Aq-EtOH	3.42 ± 0.003 ^b^	0.09 ± 0.005 ^b^	1.79 ± 0.014 ^b^
	Hex	0.48 ± 0.005 ^d^	0.05 ± 0.002 ^c^	0.23 ± 0.005 ^d^
	Et2O	0.79 ± 0.006 ^c^	0.05 ± 0.000 ^c^	0.35 ± 0.019 ^c^
*C. cibarius*	Aq	0.79 ± 0.006 ^a^	0.03 ± 0.000 ^c^	1.12 ± 0.005 ^a^
	Aq-EtOH	0.66 ± 0.006 ^b^	0.02 ± 0.000 ^d^	1.00 ± 0.003 ^b^
	Hex	0.58 ± 0.003 ^c^	0.28 ±0.005 ^b^	0.28 ± 0.009 ^d^
	Et2O	0.29 ± 0.005 ^d^	0.30 ± 0.000 ^a^	0.37 ± 0.005 ^c^

TPC-total phenolic content; TFC-total flavonoid content; TEAC-Trolox equivalent antioxidant capacity; dw-dry weight; Aq-aqueous extract; Aq-EtOH-hydro-alcoholic extract; Hex-hexanic extract; Et2O-etheric extract. Values are expressed as mean ± standard deviation of three replicates. Different letters in the same column indicate a statistically significant difference at *p* < 0.05 (Tukey’s test).

**Table 2:** Existing literature as data on *B. edulis* and *C. cibarius* mushroom extracts.

Mushroom Species	Type of Sample	Type of Solvent Used	TPC	TFC	Antioxidant Activity by DPPH Assay	Reference
*B. edulis*	Lyophilized mushrooms	Pure methanol	5.03 mg GAE/g	1.75 mg CAE/g	-	[23]
	Air-dried mushrooms	Pure methanol	31.64 mg GAE/g extract	0.458 mg QUE/g extract	80.15% at an extract concentration of 1 mg/mL	[24]
	Freeze-dried mushrooms powder	Pure methanol	≈5.5 mg GAE/g	≈2.0 mg CAE/g	-	[9]
	Air-dried mushrooms powder (93.5% dry matter)	80% Methanol	12.78 mg GAE/g	-	93.18% 3.95 mg/mL EC_50_	[20]
	Freeze-dried mushrooms powder	Pure methanol	8.0 mg GAE/g dw for *B. edulis* f. *pinicola* 7.3 mg GAE/g dw for *B. edulis* f. *beticola*	0.13 mg QUE/g dw for *B. edulis* f. *pinicola* 0.13 mg QUE/g dw for *B. edulis* f. *beticola*	≈79% for *B. edulis* f. *pinicola* ≈60% for *B. edulis* f. *beticola*	[26]
		Water	12.5 mg GAE/g dw for *B. edulis* f. *pinicola* 11.2 mg GAE/g dw for *B. edulis* f. *beticola*	0.37 mg QUE/g dw for *B. edulis* f. *pinicola* 0.33 mg QUE/g dw for *B. edulis* f. *beticola*	≈81% for *B. edulis* f. *pinicola* ≈79% for *B. edulis* f. *beticola*	
	Lyophilized mushrooms powder	Acetone/water (4:1, *v*/*v*)	28.56 mg GAE/g extract	-	0.43 mg/mL EC_50_	[34]
	Air dried mushrooms (at 25 °C for 15 days)	70% Ethanol	21.32 mg GAE/g extract	116.64 mg QUE/g extract	0.62 mg/mL IC_50_	[10]
		70% Methanol	18.96 mg GAE/g extract	97.5 mg QUE/g extract	0.73 mg/mL IC_50_	
		Hot water	17.22 mg GAE/g extract	82.85 mg QUE/g extract	0.57 mg/mL IC_50_	
		Cold water	15.98 mg GAE/g extract	61.42 mg QUE/g extract	0.66 mg/mL IC_50_	
	Dried mushrooms powder	80% Ethanol	35.56 mg GAE/g	-	87.74% RSA	[16]
	Air-dried in the shade mushrooms powder	96% Ethanol	35.83 mg/g	-	411.63 µg/mL EC_50_	[18]
		Methanol/water (1:1, *v*/*v*)	72.78 mg/g	-	151.44 µg/mL EC_50_	
*C. cibarius*	Lyophilized mushrooms	Pure methanol	0.88 mg GAE/g	0.67 mg CAE/g	-	[23]
	Dried mushrooms powder	Pure methanol	1.75 mg GAE/g extract	-	19.65 mg/mL EC_50_	[25]
	Freeze-dried mushrooms powder	Pure methanol	≈2.0 mg GAE/g	~ 1.5 mg CAE/g	-	[9]
	Fresh mushrooms (6.5% dry matter)	Pure methanol	11.94 mg GAE/g	1.72 mg CAE/g	-	[3]
		Water	-	-	-	
	Lyophilized mushrooms powder	2 M Hydrochloric acid solution	-	-	-	[38]
	Air-dried in the shade mushrooms powder	96% Ethanol	11.27 mg/g	-	>833.34 µg/mL EC_50_	[18]
		Methanol/water (1:1, *v*/*v*)	11.53 mg/g	-	730.37 µg/mL EC_50_	
	Dried mushrooms (98.2% dry matter)	Acetone/water/acetic acid (70:29.5:0.5, *v*/*v*/*v*)	3.20 mg GAE/g	0.35 mg CAE/g	10.92 µmol TE/g	[35]
	Dried mushrooms powder (at 60 °C)	60% Methanol	1.41 mg GAE/g dw	0.27 mg CAE/g dw	64.10% RSA	[19]

TPC-total phenolic content; TFC-total flavonoid content; DPPH radical scavenging activity-radical scavenging activity; dw-dry weight; CAE-catechin equivalents; EC_50_-half maximal effective concentration; IC_50_-half-maximal inhibitory concentration.

**Table 3:** Content of polyphenolic compounds (µg/g dw) in *B. edulis* extracts.

Crt. No.	Compound	Chemical Class	Chemical Subclass	Aq	Aq-EtOH	Hex	Et2O
1	2,4-Dihydroxybenzoic acid	PAs	HBA_S_	590.70 ± 2.842 ^a^	177.96 ± 2.210 ^b^	0.62 ± 0.006 ^c^	5.27 ± 0.063 ^c^
2	Gallic acid	PAs	HBA_S_	371.46 ± 3.157 ^a^	235.06 ± 3.160 ^b^	0.75 ± 0.017 ^c^	2.08 ± 0.003 ^c^
3	Syringic acid	PAs	HBA_S_	934.19 ± 3.161 ^a^	578.51 ± 3.476 ^b^	0.35 ± 0.006 ^c^	8.65 ± 0.063 ^c^
4	Protocatechuic acid 4-*O*-glucoside	PAs	HBA_S_	1735.35 ± 3.789 ^a^	1603.44 ± 3.473 ^b^	7.92 ± 0.025 ^d^	18.95 ± 0.035 ^c^
5	1-*O*-Galloyl-*β*-D-glucose	PAs	HBA_S_	82.88 ± 0.205 ^a^	42.65 ± 0.221 ^b^	n.d.	n.d.
6	*p*-Hydroxybenzoic acid 4-*O*-glucoside	PAs	HBA_S_	129.31 ± 0.339 ^b^	132.48 ± 0.316 ^a^	n.d.	n.d.
7	Apigenin 7-*O*-glucoside	FVs	FVes	82.05 ± 0.196 ^a^	60.16 ± 0.189 ^b^	n.d.	n.d.
8	*p*-Hydroxybenzoic acid	PAs	HBA_S_	51.62 ± 0.079 ^a^	49.55 ± 0.063 ^b^	n.d.	n.d.
9	Catechin	FVs	FVals	122.48 ± 0.319 ^a^	95.78 ± 0.297 ^b^	n.d.	n.d.
10	Isorhamnetin-3-*O*-glucoside	FVs	FVols	70.86 ± 0.196 ^a^	61.98 ± 0.189 ^b^	n.d.	n.d.
11	Quercetin 3-*O*-acetyl-rhamnoside	FVs	FVols	76.49 ± 0.194 ^a^	45.90 ± 0.221 ^b^	n.d.	n.d.
12	Epicatechin	FVs	FVals	74.07 ± 0.221 ^b^	95.32 ± 1.894 ^a^	n.d.	n.d.
13	Myricetin 3-*O*-glucoside	FVs	FVols	83.70 ± 0.189 ^a^	76.81 ± 0.221 ^b^	n.d.	n.d.
14	Rutin	FVs	FVols	41.34 ± 0.205 ^a^	30.98 ± 0.221 ^b^	n.d.	n.d.
15	Quercetin 3-*O*-malonyl-glucoside	FVs	FVols	25.10 ± 0.126 ^a^	23.27 ± 0.079 ^b^	n.d.	n.d.
16	Quercetin 3-*O*-glucosyl-rhamnosyl-glucoside	FVs	FVols	6.12 ± 0.032 ^a^	5.87 ± 0.063 ^b^	n.d.	n.d.
17	*trans*-Cinnamic acid	PAs	HCA_S_	154.68 ± 0.316 ^a^	85.28 ± 0.191 ^b^	39.71 ± 0.158 ^d^	54.45 ± 0.065 ^c^
Total content				4632.38	3400.99	49.35	89.40

dw-dry weight; PAs-phenolic acids; FVs-flavonoids; HBAs-hydroxybenzoic acids; FVes-flavones; FVals-flavanols; FVols-flavonols; HCAs-hydroxycinnamic acids; Aq-aqueous extract; Aq-EtOH-hydro-alcoholic extract; Hex-hexanic extract; Et2O-etheric extract; n.d.-not detected. Values are expressed as mean ± standard deviation of three replicates. Different letters in the same row indicate a statistically significant difference at *p* < 0.05 (Tukey’s test).

**Table 4:** Content of polyphenolic compounds (µg/g dw) in *C. cibarius* extracts.

Crt. No.	Compound	Chemical Class	Chemical Subclass	Aq	Aq-EtOH	Hex	Et2O
1	2,4-Dihydroxybenzoic acid	PAs	HBA_S_	202.48 ± 2.217 ^a^	90.33 ± 0.322 ^b^	0.69 ± 0.006 ^c^	1.217 ± 0.016 ^c^
2	Gallic acid	PAs	HBA_S_	112.51 ± 0.319 ^a^	63.37 ± 0.101 ^b^	n.d.	0.286 ±0.025 ^c^
3	Syringic acid	PAs	HBA_S_	166.69 ± 1.895 ^a^	171.60 ± 1.901 ^a^	n.d.	0.087 ± 0.006 ^b^
4	Protocatechuic acid 4-*O*-glucoside	PAs	HBA_S_	179.90 ± 1.932^b^	253.54 ± 0.316 ^a^	5.40 ± 0.032 ^d^	20.67 ± 0.095 ^c^
5	1-*O*-Galloyl-*β*-D-glucose	PAs	HBA_S_	14.43 ± 0.063 ^a^	7.72 ± 0.028 ^b^	n.d.	n.d.
6	*p*-Hydroxybenzoic acid 4-*O*-glucoside	PAs	HBA_S_	48.76 ± 0.095 ^a^	23.20 ± 0.095 ^b^	n.d.	n.d.
7	*p*-Hydroxybenzoic acid	PAs	HBA_S_	25.98 ± 0.126 ^b^	67.15 ± 0.221 ^a^	n.d.	n.d.
8	Catechin	FVs	FVals	111.94 ± 0.341 ^b^	115.46 ± 0.357 ^a^	n.d.	n.d.
9	Quercetin 3-*O*-acetyl-rhamnoside	FVs	FVols	12.75 ± 0.028 ^b^	18.14 ± 0.063 ^a^	n.d.	n.d.
10	Epicatechin	FVs	FVals	153.81 ± 0.253 ^b^	194.39 ± 2.210 ^b^	n.d.	n.d.
11	Myricetin 3-*O*-glucoside	FVs	FVols	12.00 ± 0.022 ^a^	6.12 ± 0.028 ^b^	n.d.	n.d.
12	Rutin	FVs	FVols	27.00 ± 0.189 ^a^	22.03 ± 0.025 ^b^	n.d.	n.d.
13	Quercetin 3-*O*-malonyl-glucoside	FVs	FVols	8.94 ± 0.022 ^a^	6.94 ± 0.021 ^b^	n.d.	n.d.
14	Quercetin 3-*O*-glucosyl-rhamnosyl-glucoside	FVs	FVols	11.51 ± 0.025 ^b^	22.03 ± 0.032 ^a^	n.d.	n.d.
Total content				1088.69	1062.02	6.09	22.26

dw-dry weight; PAs-phenolic acids; FVs-flavonoids; HBAs-hydroxybenzoic acids; FVes-flavones; FVals-flavanols; FVols-flavonols; HCAs-hydroxycinnamic acids; Aq-aqueous extract; Aq-EtOH-hydro-alcoholic extract; Hex-hexanic extract; Et2O-etheric extract; n.d.-not detected. Values are expressed as mean ± standard deviation of three replicates. Different letters in the same row indicate a statistically significant difference at *p* < 0.05 (Tukey’s test).

## Data Availability

Not applicable.

## Sample Availability

Samples of the mushroom powders are available from the authors.
